# Inflammatory Arthritis, Sacroiliitis, and Morphea: Evidence of a Systemic Inflammatory Disease

**DOI:** 10.1155/2013/347694

**Published:** 2013-08-07

**Authors:** Mohammed A. Omair, Sindhu R. Johnson

**Affiliations:** ^1^Toronto Scleroderma Program, Division of Rheumatology, Department of Medicine, Toronto Western Hospital, 399 Bathurst Street, Mount Sinai Hospital, University of Toronto, Toronto, ON, Canada M5T 2S8; ^2^Division of Rheumatology, Department of Medicine, King Saud University, Riyadh 11468, Saudi Arabia

## Abstract

Morphea is a skin disease characterized by local skin inflammation and fibrosis. Extracutaneous manifestations have been described with this disease including inflammatory arthritis. We describe a case of morphea who developed inflammatory polyarthritis and sacroiliitis coincident with new skin lesions.

## 1. Introduction

Morphea or localized scleroderma is an idiopathic disorder characterized by local inflammation followed by fibrotic changes in the skin and subcutaneous tissues. Joint involvement has traditionally been thought to be related to the extent of skin disease, manifesting as arthralgia, impaired joint mobility, or joint contracture [1, 2]. We describe a patient who developed inflammatory polyarthritis and sacroiliitis with worsening of her morphea.

## 2. Case

A 49-year-old female presented to the Toronto Scleroderma Program with a history of biopsy-proven circumscribed morphea over the abdomen diagnosed in 1994. At that time, her lesions improved spontaneously with no need for treatment. In 1998, after suffering a motor vehicle accident necessitating back surgery, she developed another circumscribed lesion around the surgical scar, which was managed conservatively. In 2008, the patient started to notice the development of multiple progressive linear lesions involving the upper and lower limbs with skin hardening and hyperpigmentation, but sparing the hands and feet. In June 2011, she noted progressive spreading and thickening of these lesions. Over the same time period, she developed swelling and pain involving the metacarpophalangeal (MCP), proximal interphalangeal (PIP) joints and left knee. Additionally, she developed inflammatory back pain with more than 1 hour of morning stiffness. The patient had recurrent attacks of uveitis, treated with local steroids, with no visual sequelae. The patient did not exhibit any symptoms suggestive of systemic sclerosis, systemic lupus erythematosus (SLE), or inflammatory bowel disease. Her family history was significant for psoriasis in her father. In October 2011, methotrexate 15 mg/week was initiated with subsequent improvement of her peripheral joint pain and swelling, but there was no significant response in the axial pain and stiffness. 

She was first seen in our clinic in June 2012. Her examination revealed linear morphea involving all 4 limbs with active inflammation and chronic atrophic changes in the abdominal and back areas but no psoriatic lesions. She had tenderness and swelling involving the MCP, PIP, and left knee joints with swollen and tender joint counts of 5 and 6, respectively. Axial examination revealed no abnormalities in the Schober's test, FABER's test, and finger-to-floor and occiput-to-wall distances. Her C-reactive protein was elevated at 10.1 mg/L, while antinuclear antibodies, anti-Ro, anti-La, rheumatoid factor, anti-cyclic citrullinated peptide, and HLA-B27 were negative. Magnetic resonance imaging (MRI) of the sacroiliac (SI) joints revealed sclerosis at the posterosuperior aspect of the left SI joint. Edema was seen in the middle and inferior portions of both SI joints. Erosive changes on the subchondral endplate were seen on the left side. Radiographs of the hands revealed no periarticular osteopenia, periostitis, or erosions. Ultrasound of the fingers was positive for synovial thickening and power Doppler signal in multiple PIP and MCP joints. Her methotrexate was increased to 20 mg/week.

## 3. Discussion

Localized scleroderma is generally considered a benign, self-limited condition confined to the skin and subcutaneous tissues [3]. Musculoskeletal involvement typically includes growth defects with shortening of the affected limb [1, 2, 4], scoliosis [1, 2], thorax asymmetry [1], and muscle atrophy [2]. The presence of concomitant arthritis was thought to be mechanical in etiology, largely related to contracture of the skin overlying the joint [2–5]. In patients with generalized morphea, arthritis has also been reported to occur in the context of an overlap with another seropositive disease such as SLE or rheumatoid arthritis [5]. However, this paradigm may be insufficient. Features of this case, supported by several lines of published evidence, suggest that localized scleroderma itself is a systemic inflammatory process.

In a multinational cohort of 750 children with localized scleroderma, extracutaneous manifestations have been reported. These include articular (47%), neurologic (17%), vascular (9%), and ocular (8%) involvement. In 1/4 of these patients, the articular involvement was unrelated to the site of skin lesions. Six percent developed an inflammatory oligoarthritis while 5% developed a polyarthritis. This has led to recent speculation that localized scleroderma may be a systemic, inflammatory condition [6]. Noteworthy features of this case are the presence of sacroiliitis, history of uveitis, and a family history of psoriasis. These features are more typical of seronegative rheumatic diseases but do not necessarily indicate an overlap with a seronegative disease. Morphea and spondyloarthropathy occurring concomitantly has only been described in 3 patients [5]. The presence of anterior uveitis and other inflammatory ocular disorders (episcleritis, keratitis) has been reported in observational studies of morphea patients [2–4]. The inflammatory ocular lesions can be unrelated to the site of the skin lesion [3]. Similarly, a family history of rheumatic or autoimmune disease has been reported in 10%–20% of patients with localized scleroderma [1, 5, 7]. Reported conditions include rheumatoid arthritis, systemic sclerosis, systemic lupus erythematosus, psoriasis, vitiligo and lichen sclerosis [1, 5, 7]. The frequent presence of a family history of these conditions supports the hypothesis that a genetic background may contribute to the susceptibility to clinically distinct autoimmune inflammatory diseases [7].

Most importantly in this case, her morphea activity was concordant with both her inflammatory polyarthritis and sacroiliitis. She had an elevated C-reactive protein, and she required systemic immunosuppression to treat her disease. There was no evidence of an overlap with another seropositive disease. 

This case and a review of the literature support the hypothesis that localized scleroderma is a systemic inflammatory process. This also supports the proposed shift in paradigm that localized scleroderma and systemic sclerosis are two ends of a continuous disease spectrum [3]. It is increasingly recognized that inflammatory polyarthritis can occur in systemic sclerosis [2, 8] and has a detrimental effect on quality of life [9] and that the arthritis responds to biologic agents [10]. Given that inflammatory arthritis can be destructive, lead to disability and yet is treatable, localized scleroderma patients should be identified, thoroughly evaluated, and considered for systemic immunosuppressive therapy [3].

## References

[1] Christen-Zaech S, Hakim MD, Afsar FS, Paller AS (2008). Pediatric morphea (localized scleroderma): review of 136 patients. *Journal of the American Academy of Dermatology*.

[2] Marzano AV, Menni S, Parodi A (2003). Localized scleroderma in adults and children. Clinical and laboratory investigations of 239 cases. *European Journal of Dermatology*.

[3] Zulian F, Vallongo C, Woo P (2005). Localized scleroderma in childhood is not just a skin disease. *Arthritis and Rheumatism*.

[4] Uziel Y, Krafchik BR, Silverman ED, Thorner PS, Laxer RM (1994). Localized scleroderma in childhood: a report of 30 cases. *Seminars in Arthritis and Rheumatism*.

[5] Leitenberger JJ, Cayce RL, Haley RW, Adams-Huet B, Bergstresser PR, Jacobe HT (2009). Distinct autoimmune syndromes in morphea: a review of 245 adult and pediatric cases. *Archives of Dermatology*.

[6] Zulian F, Vallongo C, Patrizi A (2012). A long-term follow-up study of methotrexate in juvenile localized scleroderma (morphea). *Journal of the American Academy of Dermatology*.

[7] Zulian F, Athreya BH, Laxer R (2006). Juvenile localized scleroderma: clinical and epidemiological features in 750 children. An international study. *Rheumatology*.

[8] Low AHL, Lax M, Johnson SR, Lee P (2009). Magnetic resonance imaging of the hand in systemic sclerosis. *Journal of Rheumatology*.

[9] Johnson SR, Gladman DD, Schentag CT, Lee P (2006). Quality of life and functional status in systemic sclerosis compared to other rheumatic diseases. *Journal of Rheumatology*.

[10] Phumethum V, Jamal S, Johnson SR (2011). Biologic therapy for systemic sclerosis: a systematic review. *Journal of Rheumatology*.

